# Forensic Medicine.—Duration of Pregnancy

**Published:** 1839

**Authors:** 


### FORENSIC MEDICINE.—DURATION OF PREGNANCY.

### CASE OF THE REV. FERGUS JARDINE.

RECORD OF THE PROCEEDINGS IN THE REFERENCE BY THE SYNOD OF FIFE, TO THE VENERABLE THE GENERAL ASSEMBLY OF THE CHURCH OF SCOTLAND, IN MAY 1839, OF THE CASE OF THE PARISHIONERS OF KINGHORN against THE REVEREND FERGUS JARDINE. Edinburgh, 1839. pp. 178.

THE above document having come into our hands through the kindness of a reverend member of the venerable assembly by which final judgment has been awarded in this interesting case, we gladly avail ourselves of the opportunity to lay the case, with the evidence and judgment, before the medical public.

In doing so, we shall not anticipate the conclusions which our readers may draw from all the facts stated, by any prefatory observations of our own, upon a subject confessedly one of the most difficult with which the medical jurist has to do.

### I.—LEGAL HISTORY OF THE CASE.

On the 3rd of March, 1835, the Rev. Fergus Jardine, minister of Kinghorn, was married to Janet, daughter of the Rev. Dr. Martin, minister of Kirkcaldy. On the 24th of August following, the lady was delivered of a living daughter, whose existence was protracted till the 20th of March, 1836, when she expired.

At a meeting of the Presbytery of Kirkcaldy, the 2nd of September, 1835, Dr. Martin, the infant's maternal grandfather, stated to the members "that his daughter, Mrs. Jardine, had brought forth a premature child, within the sixth month from the date of their marriage; and as that child, though both feeble and extraordinarily diminutive, still lived, he had been informed that reports most injurious to the character of both parents, and most prejudicial to the reputation and usefulness of Mr. Jardine in particular, were in general circulation; that it was therefore essential to the character of the Presbytery itself, as well as of Mr. and Mrs. Jardine, that that matter should be inquired into, and that the opinion of some competent professional man should be taken, whether the state and appearance of that child was such as to be consistent with its being the legitimate fruit of the marriage of its parents. He stated, that Dr. Reid, of Kirkcaldy, by whom Mrs. Jardine had been delivered, was willing to declare to the Presbytery all that he knew of the case, but that he felt difficulted to pronounce an opinion upon it, because it was one to which nothing similar had ever occurred to him before; that besides, it was not probable that any opinion given by Dr. Reid would be satisfactory to the public, however favourable it might be, on account of his personal connexion with the case; that it would be expedient that one of the most distinguished practitioners in puerperal cases should be requested to visit and inspect the child, to inquire into all the circumstances which he might deem essential, and to report his opinion to the Presbytery; that it was more desirable that this should be done by the authority and at the request of the Presbytery, than at that of Mr. Jardine himself.

Mr. Jardine made a statement to the same effect, and mentioned, that he had some reason to think that the injurious *fama* so confidently circulated, had, in a great measure, taken its rise from the child of Mrs. Brodie, Mrs. Jardine's sister, which was eight weeks old, having been seen by some persons in the arms of a servant at the manse of Kinghorn, where Mrs. Brodie had arrived on the day of Mrs. Jardine's delivery, and where she had remained three days, to afford the nourishment of her milk to the prematurely born infant; and that those persons, being ignorant that there was any other child in the house, had supposed Mrs. Brodie's child to be her sister's, and, consequently, a child, not only fully mature, but remarkably well-grown. He joined in requesting the meeting to take steps for the investigation of the case, that an end might be put to reports at once so painful to him, and so hurtful to his usefulness as a minister.

The meeting unanimously concurred in the expediency of these suggestions, and authorised the Moderator to write to Dr. Hamilton, Professor of Midwifery in the University of Edinburgh; and, if he should decline coming to Fife, or should be othewise engaged, to Dr. Thatcher, Professor of Midwifery to the Royal College of Surgeons in the same city, requesting him to inquire into, and report upon, the case.

The said letter was written forthwith, and transmitted by the post of the same evening. On the 4th, Dr. J. Hamilton, jun. replied by two letters, in the first of which, he regretted his inability, because of prior engagements, to leave Edinburgh for two hours;—stated his experience to have been, that no infant was *reared to maturity*, who was born earlier than seven calendar months, although he had "repeatedly seen infants born within a week of six calendar months, live for days, swallow readily, and pass urine and fæces." One of whom he could swear to having lived completely five days.

Two cases are cited in his letters from other authorities, one Dr. Rodman's, of Paisley, published in the 11th volume of the Edinburgh Medical and Surgical Journal, of an infant born (of parents who had had a family, and where there could be no inducement to mistake or misrepresent) nineteen weeks after conception, and reared to a certain age. The other, that of the Rev. T. Elder, minister of Whitehorn, where the infant born, was so within five months after marriage, and lived for some months at least. The father, here, had been deposed in consequence, but was subsequently reinstated in his clerical status, partly on account of his personal character, and partly on the faith of a statement made by Dr. Pitcairn, then, perhaps, equal in celebrity to any physician in Europe, and by Drs. Preston and Drummond, that it was consistent with their observation and experience that "a child born in the beginning of the *sixth* lunar month, may be alive and continue in life."

Dr. Hamilton not undertaking the inquiry, Dr. Thatcher was instantly written to, and on the 12th instant he examined the child, and took the evidence of Dr. Reid the accoucheur; Mrs. Robertson, who had been present at the birth; and the nurse who had had charge of the child from birth up to that date.

On the 15th of September the Presbytery met, to consider the above correspondence, and the result of the medical examination. A certificate and a note from Dr. Thatcher, were also laid before it. The certificate simply expressing his opinion that the child was "still of the premature character, and must have been *decidedly more so, in every respect, at birth.*" An opinion formed upon the history of the case,—its (the child's) augmented development since birth, and every point indicative of immaturity. The note further stated, that, from a full consideration of the evidence and other points, the Doctor felt bound to report that this child was "among the '*cas rares*' which occur of being prematurely born, and of *vitality being afterward extended by the most extreme care and attention.*"

This certificate and note, with the letters of Dr. Hamilton, removed, in the judgment of several members, all suspicion of immoral conduct from Mr. Jardine, and all cause for farther inquiry. To others it appeared desirable that the declaration of Dr. Thatcher should have been more explicit in regard to the probability or possibility of the child having been procreated within the time of her father's marriage; and he, being again written to, for a more explicit answer on this point, replied in the affirmative.

On the 23d of September, his second certificate was laid before the Presbytery, which, after deliberation, unanimously agreed that there was no ground to charge Mr. Jardine with the criminality alleged against him.

Here the matter might have terminated, but for the malevolent and persecuting spirit of a few dissatisfied men, parishioners of Kinghorn, but neither ministers nor members of Presbytery, who, under the name of petitioners, had previously requested the Presbytery to hold a "visitation" at Kinghorn, before coming to any decision, and who now protested against the finding, and appealed to the Synod of Fife.

The *animus* of the petitioners is evident from the "reasons of appeal" lodged by them, and which we give seriatim. They are as follow:—

"1. Because the petition for the parishioners of Kinghorn ought to have been considered and decided upon before proceeding to give any determination on the matter at issue. 2. Because, after receiving the petition of the parishioners of Kinghorn, praying the Presbytery to make a presbyterial visitation of the parish, &c., before proceeding to decide in the case, and resolving to take it into consideration at the next ordinary meeting; it was irregular and incompetent in the pro re nater meeting to come to any determination on the merits of the case, whereby the petition was virtually refused. 3. Because the Presbytery proceeded to determine upon secondary evidence, brought from a distance, when primary evidence could have been obtained on the spot. 4. Because the medical evidence, on which the Presbytery decided, was not given on soul and conscience; and, in particular, because Dr. Thatcher has not acted strictly in his professional capacity, but, before entering upon the requisite investigation, stated, that he trusted it would be in his power satisfactorily and happily to arrange the case. 5. Because the evidence of Dr. Hamilton, jun. who was first applied to, goes to establish, that a child born before the expiry of seven calendar months from conception, cannot live.

6. Because the *fama clamosa* mentioned in the petition having special reference to the question before the Presbytery, it ought to have been inquired into before coming to any determination."

The answers for the Presbytery of Kirkcaldy, to the "*reasons*" of those unreasonable men who, under the guise of petitioners, were thus manifesting the rancour of persecutors, are grave, dignified, and so admirable that we cannot forbear giving them a far wider circulation than they would otherwise obtain.

"1. To the first of those reasons it may be enough to reply, that it merely asserts that the members of the Presbytery convened at the said meeting, instead of acting according to their own judgment, ought to have been guided by that of the petitioners, an obligation to which, they are not disposed to admit.

2. To the second, it needs only to be answered, that it assumes that, because a petition was presented to the meeting, and received, so as to be laid on the table, and to be, if necessary, the subject of future consideration, therefore the Presbytery were bound forthwith to grant the whole prayer of it. A petition may be received and laid on the table, from respect to those by whom it is presented, while yet its prayer may be refused in part or altogether. To say otherwise were just to say, that so soon as a petition is laid before a Presbytery, and is allowed by them to lie on their table, they are thenceforward to have no mind of their own, or power to act, as to any matter referred to in such petition. As to the irregularity or incompetency alleged in this reason, it is replied, that if it was competent for the Presbytery, at such a meeting, to receive the petition in question, it was without doubt equally competent for them to dispose of it as they thought proper. But though it is obviously altogether needless to say more in answer to these reasons, the members of the Presbytery may further observe, that after being at considerable pains to obtain the best medical evidence which they could procure, that evidence which alone could be satisfactory in such a case, they had met to consider and determine upon it. And where could be the reason or justice of refusing to decide upon that evidence, because a petition, never presented till then, stated that certain reports had been in circulation, reports, too, of the tenor of which nothing was specified. Several members of Presbytery well knew that very injurious reports had been in circulation. That was the cause of their repeated meetings p. r. n. and they knew that the facts of the premature birth and continued life of Mr. Jardine's child were the basis of these reports. And now, when the Presbytery had obtained that evidence, by which alone a satisfactory judgment could be formed in regard to these facts, they are told by the petitioners that they are not to judge of that evidence. Whatever the reports of which the petitioners speak may be, they cannot affect the evidence in question. If they have other evidence to bring, they are at perfect liberty to produce it, and if relevant, it will be judged of. The deliverance of the meeting p. r. n. appealed from, is on the medical evidence before it, and on no other, and that evidence, it is clear that no examination into the alleged reports could alter.

3. As to the complaint implied in the third reason, that the Presbytery determined upon secondary and distant evidence, when they had primary evidence on the spot,—if the Presbytery understand those expressions, it is that they preferred employing Dr. Thatcher in examining Dr. Reid and others, and in judging of their evidence, to examining them and judging of their evidence themselves. They were not competent either to make the examination or to judge of the evidence obtained. Though they had taken all the evidence which Dr. Reid and others could give, they must, after all, have submitted it to the judgment of some such person as Dr. Thatcher; and it was manifestly far better that he, who was so much more competent to determine what questions ought to be put, and what opinion to form from the replies, than they could be, should examine for himself. And in Dr. Thatcher's certificate and notes, they have the result of Dr. Reid's evidence, as given by him 'on soul and conscience, in truth,' as well as of the evidence of the other witnesses. Dr. Thatcher's evidence, moreover, was evidence '*on the spot*,' for he came to the manse of Kinghorn personally, examined the condition both of the infant and its mother, and there also conversed at length with Dr. Reid, and the other witnesses mentioned in his note.

4. Of the fourth reason, it is not easy to speak without some feeling of indignation. However guarded in its expression, it has no meaning, if it do not imply an insinuation against the integrity of Dr. Thatcher; that he had acted as a partisan, and not as a man of truth and honour. They object that he has not used the asseveration 'on soul and conscience.' But these words do not differ in import from those which the petitioners or their agents know that he did use; for they were in possession of full copies of his communications with the modera-tor and clerk of the Presbytery; in the first of which, that of the 7th Sept., he says, 'I shall be most happy, *conscientiously and scientifically*, to investigate the case you mention,' and in another of which, his note of the 12th September, he states, 'I considered it my duty to approach this investigation in the *most con-scientious and guarded manner!*' Yet, with these words before them, they in-sinuate that he did not act with a regard to conscience. But the petitioners, or the person who drew out the reasons given in their name, insinuate farther, that a proof of partiality is found in the hope expressed by the Doctor, where he says, 'I trust I shall be able satisfactorily and happily to arrange this case.' Now as to these words, it is to be observed, that they express nothing as to the nature of the conclusion at which he hoped to arrive, farther than that he trusted it would be satisfactory, we may suppose, to those who consulted him, and happy, in so far perhaps, as it would enable them to decide without hesitation. But suppose it did express, what certainly it does not, a trust, or even a wish, that the issue might be happy for Mr. Jardine, the Presbytery ask, how else could any good man have expressed himself, if he chose to express any hope on the subject; any man who has a regard to the interests of morality and religion, must be aware of the injury likely to be sustained by those interests, should a minister of the gospel be proved guilty of a crime imputed to Mr. Jardine, and the Doctor believing him innocent so long as he knew no cause to hold him guilty, most naturally expressed the hope referred to. He must have been both a bad and an uncharitable man, had he thought or spoken otherwise; and is it to be insinuated, that, because he did not, a man of his eminent reputation, and conspicuous situation, had wilfully violated his honour, and cast away his character.

5. What is affirmed in the fifth reason, as to what is termed Dr. Hamilton's evidence, *is not true*. The Doctor takes care to limit his statement to what had fallen under his own observation, and to prevent the unfavourable conclusion which might be drawn from it, he subjoins the remarkable case detailed by Dr. Rodman. And, to what was it owing, that, in the evening of the same day, he wrote another letter, communicating the recorded opinion of Drs. Pitcairn, Preston, and Drummond? to what but his anxiety, lest what he had given as the result of his own observations in practice, should lead to an inculpation of Mr. Jardine, which he did not intend? The case of Mr. J's child is undoubtedly a rare one, *un cas rare*, as it is expressed by Dr. Thatcher, though by no means unprecedented. And the Presbytery affirm, that even had there been nothing before them but the letters of Dr. Hamilton, these, taken in connection with the weight and appearance of the child, and other circumstances, would have made it imperative on them to acquit their brother of the criminality with which he had been charged; for, if it be proved, in but a single instance, that such a case has been, it is nothing more than justice to parties, hitherto unimpeached, to admit that their's may be similar. How much more, then, when Professor Thatcher declares, that he considers the *fama* on this circumstance, against the minister of Kinghorn, as altogether groundless.

6. As to the sixth reason, it is sufficient to remark, that the members of Pres-bytery surely were not ignorant that the *rumours and matters* referred to in the petition, (for which expression the words *fama clamosa* are substituted in this reason), had respect to the birth of Mr. Jardine's child. Why else these repeated meetings p. r. n.? Why else their inquiries?"

The Synod very properly dismissed the appeal, approved of the Presby-tery's "diligence and finding," and remitted the matter "to be considered by the Presbytery at their next ordinary meeting, it being understood, in terms of their finding, that the petitioners should be then heard, if they think proper, upon their petition."

On the 28th of October the Presbytery again met. Dr. Martin, the father of Mrs. Jardine, declined to act as clerk, and both he and Mr. Jardine declined sitting in their capacity of judges, the latter sisting himself as a party at the bar. Two motions were then put, the 1st, That in compliance with the wishes of the petitioners, a presbyterial visitation should be held at Kinghorn. The 2nd, that the prayer of the petitioners for a visitation be refused, but that it be left to them to serve Mr. Jardine with a libel, if they shall see cause. The first was carried by the casting vote of the moderator, Dr. Martin having refused to vote.

Against this finding the Rev. plaintiff appealed to the Synod, alleging as his reasons,—1st. That no grounds were laid in the petition calling for or justifying the proposed visitation of his parish. 2ndly. That it was contrary to ecclesiastical law and practice, to subject a minister and his parish to presbyterial interference, where the former is merely accused of a single and specific offence. 3rdly. That such visitation would retard rather than forward the inquiry. 4thly. That the sentence appealed from, *in effect* enabled the petitioners "to lead evidence against the appellant, on a criminal charge, which, if well founded, would infer his deposition before any libel has been raised against him, and before he has been in any way made aware either of the allegations in detail, which are to form the subject of proof, or of the witnesses by whom it is proposed to establish them." 5thly. That the Presbytery having found, and the Synod affirmed their finding, after due enquiry, that there was no ground to charge him with the criminality alleged against him, "it is incompetent, unconstitutional and unjust, to entertain farther procedure in the matter in any other form than by libel, to which the petitioners have it in their power to resort at any time. And 6thly. That the deliverance of the Presbytery ought to have been a simple refusal of the petition, reserving to the petitioners to libel the appellant if they should see cause, or be so advised.

The Synod met on the 12th of April, 1836, and "unanimously agreed to sustain the appeal, reverse the sentence of the Presbytery of Kirkcaldy, appointing a presbyterial visitation of the parish of Kinghorn. Remit the case to the Presbytery, with instructions to make the fullest investigation into the grounds of the *fama* against the minister of Kinghorn, by summoning witnesses, and taking a precognition in the usual form, allowing the minister and the petitioners to be fully heard, and to proceed by libel, if they shall see cause, according to the rules of the church." The appellant and the Presbytery acquiesced in this judgment—but the petitioners made a show of more vexatious litigation by an appeal to the ensuing General Assembly, which appeal, however, they failed to prosecute. And on the 11th and 16th of the following May the Presbytery resumed their sittings on the case; at the last of these meetings, a precognition of witnesses was determined on, and a committee of the whole members of Presbytery appointed to conduct it. On the 5th of July, at the the request of Mr. Jardine, a limit was set to the time for precognoscing witnesses against him. At another meeting, on the 3rd of August, the impudent, barefaced, and indecent request was preferred by a Mr. Thomas Barclay, "in his own name, and in that of the other petitioners," "that Mr. and Mrs. Jardine, the parties charged with the guilt alleged in the petition, should be judicially examined, and their declarations taken." The request was very properly refused, whereupon Mr. Thomas Barclay protested and appealed to the ensuing Synod —which met on the 11th of October, dismissed the appeal, and affirmed the sentence of the Presbytery. The judgment of the Synod was as unpalatable to these self-constituted conservators of public morals, as the previous sentence of Presbytery, and they appealed from it to the ultimatum of the General Assembly. And, on the 30th of May, 1837, accordingly, two years after the marriage, and eighteen months from the commencement of this vexatious and harassing inquiry, the case came before the Assembly on the preliminary point thus raised. That venerable body, however, dismissed the appeal, and remitted the matter to the Presbytery of Kirkcaldy with instructions to sist all further procedure, and to dismiss the cause, "unless a libel shall be laid on the table of the Presbytery by the first day of the following September."

This decisive style, reduced the petitioners to the alternative of framing a libel against their minister, or abandoning the persecution to which they were subjecting him. They made their election of the former—and proposed to prove their allegations by not less than 38 witnesses, including Drs. James Hamilton, Thomas Stewart Traill, Wm. Campbell, and Peter Fairbairne, and Surgeons Reid, Storrer, and Zeigler. The medical evidence both pro and con we shall presently analyse, but for the present waive the consideration in it, in order to complete the legal history of the proceedings of this case.

On the 1st of Sept. 1837, the "libel" was laid on the table of the Presbytery, indicting and accusing the Rev. Fergus Jardine, of antenuptial fornication, alleged to have been committed by him in Dec. 1834, "and on each, or one or other of the first two days of the month of March, 1835; and containing several allegations in confirmation of the same. On the 8th of September the Presbytery resolved to serve the Rev. Gent. with said libel, and the list of witnesses, and to cite him to compear before an ordinary meeting of Presbytery, on the 4th of October, on which day he appeared for himself and gave in written answers to the libel." "After deliberation the said libel was found relevant. Dr. Laird, Mr. Brewster, and Mr. Sieveright were appointed to withdraw with Mr. Jardine, for the purpose of dealing with him in *foro conscientiæ*, concerning the charges brought against him by the libellers. Dr. Laird, as oldest member of the committee appointed to converse with Mr. Jardine, now reported, that having solemnly asked the accused, as in the presence of God, and in the view of the great day of Judgment, whether he had been guilty of one or all of the offences libelled? the said committee were answered by Mr. Jardine, that, bearing in mind those awful considerations, he declared his entire innocence of one and all of those charges."

On the 30th the Presbytery again met for probation—when Mr. Deas, the advocate for the libellers, requested that, as the present matter depended to a considerable extent upon medical evidence, the medical evidence from Edinburgh might be present during the whole of the evidence, except of two witnesses, in order that they might give their opinion. This was objected to by the counsel for the defender, except when the evidence bore on the medical question of gestation. The request was allowed by the Presbytery, with a limitation that they be made to retire whenever the Presbytery may deem it necessary.

The examination of witnesses for the prosecution was continued for five days, when objection was taken by the defender's counsel to Dr. Campbell, as a disqualified and inadmissible witness, but repelled by the Presbytery. Whereupon the counsel appealed to the Synod, and the Presbytery adjourned to the 18th of November, when they again met, and instructed two of their members to answer the appeal—their answers were not, however, presented until the 15th of the following March, and were then introduced with the expression of an opinion that the appeal had only been made to effect delay; and couched in such strong terms, that the appeal was departed from, and on the following day the Doctor's examination was proceeded with. On the 17th the pursuers closed their case, and, time being allowed the defenders to prepare lists of witnesses, the case for the defence was ordered to come on on the 1st of May. Seventy-nine witnesses were cited on behalf of the defender, including Drs. Thatchers, Aitkin, Christison, M'Whirter, Combe, Cummin, Alison, Reid, and Robert Collins, of Edinburgh, Glasgow, and Dublin. Drs. Dalen of Rotterdam; Pignal and Saintoné, of Paris; Chatron, of Turin; Sinibald, of Rome; and T. B. Fabbin, and Cini Annibal of Boulogne. On the 3rd of May the Presbytery adjourned to the 4th of June, and from that date to the 24th, after which a further adjournment took place to the 6th of November, and on the following day sentence was passed, finding the charge of fornication *not proven*, and further, "that the testimony of the several witnesses, both with respect to matters of fact, viz. the appearance of the child at birth, &c., and also with respect to the opinions of medical men, regarding the viability of such a premature child as the child in question is said to be, is of such an opposite and contradictory nature, that the Presbytery, with their present light, have great difficulty in coming to any decision on these points. The Presbytery, therefore, agreeably to a common maxim of law : Satius est impunitum relinqui facinus nocentis quam innocentem damnari, find the libel not proven." Against this deliverance both parties protested and appealed to the next meeting of the Provincial Synod of Fife.

We subjoin the reasons of appeal of both parties with the answers of the Presbytery thereto, so arranged as to present the whole to our readers at one view.

| Pursuers' Reasons of Appeal. | Answers for the Presbytery of Kirkcaldy, 1838. |
|---|---|
| "1. Because the evidence adduced was such as to leave no reasonable doubt that a viable child cannot be produced at so short a period of gestation as five calendar months and twenty-one days, being the alleged period of gestation of the child mentioned in the libel. | "The first reason 'is a simple assertion, and must be met with a counter assertion, that the evidence is not such, &c. or by going into the whole evidence of both sides of the case. |
| 2. Because, at all events, there could be no reasonable doubt on the evi- | The same answer applies to the second reason, which respects the appear- |

dence adduced, and having regard to the appearances of the child, the treatment it received, and the period it lived: that the child in question was born after a longer period of gestation than that just mentioned, being the period which elapsed between the date of Mr. Jardine's marriage, and the birth of the child.

ances and treatment of the child in question.

3. Because, supposing it to be established that a child was begotten before marriage, it is clear beyond all doubt that Mr. Jardine was the father of the child.

The third reason would be a good ground of appeal, provided the fact which it supposes to be established was well founded.

DEFENDER'S REASONS OF APPEAL.

Because the prosecutors having failed to establish any part of the libel, the same ought to have been found not proven, and simpliciter dismissed,—the answers or defence sustained, and the appellant absolved from the whole conclusions against him." 152.

The single reason here adduced by the defender is one in form rather than in substance. He says, 'the libel ought to have been found not proven, and simpliciter dismissed, &c.' The libel has been found *not proven*, and consequently the appellant is absolved from all the conclusions of the libel in fact, if not in form." 152.

The Provincial Synod of Fife met on the 9th of April in the present year, and agreed, with the consent of both parties, to refer the whole cause simpliciter to the ensuing General Assembly.

The Assembly met in May; the result of the reference to them was the termination of Mr. Jardine's sufferings, and the completion of his triumph. They dismissed the appeal of his prosecutors, sustained his own appeal, and amended the sentence of the Presbytery by finding the libel not proven, and absolving the accused minister from all the conclusions against him.

### II.—MEDICAL HISTORY OF THE CASE.

Having traced the history of the proceedings in this case from the birth of the child to the acquittal, long after its decease, of its parents of all criminality in its production, we proceed to exhibit the several features of its medical history, as they are presented to us on the testimony of eye-witnesses.

According to the accoucheur, who, however, did not particularly examine it, the head of the child at birth was not large in proportion to the body. The posterior fontanelle and sagittal suture were considerably open. And, during labour, the parietal bones over-lapped. The eyes were partially opened and particularly small. Its general appearance was such as he considered usual in cases of premature birth. He failed to observe whether there was hair on the head or not—what was the colour of the skin,—what the situation of the navel,—or what the state of the genitals.

According to one of the witnesses into whose hands the child came out of the hands of the accoucheur, it cried immediately, had hair on its head; the clear and natural skin of a full-born child, without wrinkles or folds ; nails on its fingers ; voided its urine immediately ; and, though not at once, took the breast within 24 hours. But, except in the assertion that it was the smallest child she had ever seen, her testimony was contradicted by every other witness to the facts.

Mrs. Martin, the infant's grandmother, who received the child for the purpose of washing and sorting it, had never beheld such an object before! According to her—its head and shoulders were tolerably formed, but from the back downwards to the lower extremities it might be said to have no form at all, there seemed to be no joints at the ankles, and no separation whatever between the lower limbs and the feet. In particular, it had no heels, but the limbs and feet were continuous and ran out like sticks.

Its colour was reddish and unnatural. Its skin generally wrinkled.

Another of the witnesses described its face as being shrivelled like a piece of chamois leather, and covered with a silky sort of down, like mould—this was after it had been washed—the skin, as generally wrinkled, and not full-filled.

A third alleged that its skin was all wrinkled together, of an indescribable brownish, reddish, unnatural colour.

A fourth, the wet-nurse, deposed that its skin was of a dark-colour, very like an old person's, all wrinkled and not well filled, with a down over the body. According to this witness it had the gum upon it at first and that continued for three weeks. But over the genitals the skin was wholly wanting.

The testimony to the state of the nails is somewhat conflicting. Mrs. Robertson and the husband of Mrs. Brodie affirming, and Mrs. Hill denying, their presence; while Mrs. Martin, Mrs. Brodie and Miss Martin, described what the two former witnesses took for nails, as some fleshy-like thing upon the fingers but not upon the toes, corresponding to nails in appearance, but differing in reality, being a sort of white skin, quite soft although harder than the skin on the other parts of the fingers, and generally ragged round the edges.

Shortly after birth it gave a kind of cry, but not at all that of an ordinary child, it was so very feeble. In the words of another witness, Mrs. Jardine's unmarried sister, its cry was a weak, squeeking, peeping cry, not at all the cry of a child come to its full time at birth. Another sister, herself married, had no remembrance of having heard it cry at all, but said it "moaned incessantly," "which made her think every moment it would die." While a third, and altogether unexceptionable witness, Mrs. Hill, the child's wet-nurse, characterized its cry as unlike that of any other child, "a sort of *wurning* unnatural cry."

To return to the testimony of the maternal grandmother, its breathing, not only upon birth, but until death, was never like another child. It would take a breath or two, then always make a pause as it were taking a rest before it could breathe again, and appeared to have a difficulty in breathing which continued while it lived. Mrs. Brodie, her daughter, also deposed to the difficulty of respiration; as did its nurse, Mrs. Hill, who said that even after it began to notice, "it had a way of putting down its head, as if it were like to be choked with something about its breast."

Mrs. Martin was the first person who attempted to give the child any food; putting a little sugar and water upon its lips, but it did not swallow.

In the course of the day (of its birth) its future nurse was brought to the Manse, and her breast offered to the child, but it would not suck. Mrs. Hill then milked a little of her milk which was endeavoured to be dropped into the child's mouth from a spoon. Some of it might have gone down, but no effort was made to get it to swallow for fear of choking it. On the evening of the same day, Mrs. Brodie arrived at the Manse with her child, a month old, at the breast, and offered her breast to her sister's infant, but it could not suck. Mrs. Brodie, however, thought it swallowed some of the milk dropped from her breast into its mouth; the wet-nurse swore it was several weeks after birth before it took the breast and sucked, and not then like another baby.

Its bowels were never known to be moved without medicine but three times in the whole course of its life, although it appears to have voided its urine at some one part or other of the day on which it was born.

According to its nurse it slept very little, and scarcely ever more than ten minutes at a time.

The grandmother thought the child's eyes opened shortly after it was born, but solemnly declared her belief that it was born blind, the strongest light, both of the sun and of candles, when applied to its eyes for the purpose of ascertaining if it could see, "having produced no more effect upon them than it would have done upon the table."

According to its maiden aunt, Miss Anne Martin, "light produced no effect upon its eyes, until about three weeks after its birth; the impression was conveyed to the mind of its married aunt, Mrs. Brodie, that it did not see, because bright light produced no effect on the child's eyes.

The nurse went the length even of stating that it was three months old before it began to notice. "The eyes were open at times, but she thought it could not see from light producing no effect upon the eyes, and from its taking no notice when she moved her hands back and forwards in front of them."

The child was born in August, and until the following October, when Miss Martin left the Manse, it appeared to her to be almost constantly in a state of suffering and distress—moaning very much, and requiring medicine daily or every alternate day to produce motion in its bowels. And when she saw it, shortly before its death, it was still very weak and feeble, and "she particularly observed that while it could grasp it could not hold anything in its hands."

The first time the nurse saw it on its birth-day, it was very sick, and vomited all the time she was in the room. While under her care, which was day and night for four weeks, it was much pained and suffered greatly. And while it needed medicine constantly, that was with difficulty administered for fear of choking it. She was sometimes up all night with it, and could not lay it down from its being unwell.

For the first three months its growth appeared to be arrested or nearly so —and even after that, although it advanced gradually it did so but slowly.

An old lady named Hamilton, saw it a week after its birth, and had never seen a human being in life so small as it appeared to be. Its face was so much wrinkled that it was miserable to behold it. And its fingers were so very small that they seemed to her to be like knitting needles. She adds, "when the child was some months old, it became a very fine one."

James Adamson, overseer at Balmuto, and one of the elders of the parish of Kinghorn, who saw the child for the first time at its baptism, and some weeks subsequently, besides being present at its funeral—had never seen so small a child in his life; and when he had it in his arms felt glad to get clear of it, "because it was too frail for his handling." Its eyes, by his statement, were "hollow and dull in the head." It had very little movement and never laughed. The coffin in which it was laid appeared to him to be a great deal smaller than those in which his own still-born children had been buried.

Dr. Thatcher, who saw the child 19 days after its birth, at the request of the Presbytery, for the express purpose of deciding upon the points at issue, neither weighed nor measured it, in consequence of its age from birth, and because he heard it had been previously found to weigh scarcely four pounds, was of opinion that it measured about 12 or 13 inches; "but, from the report of the nurse, it had assumed a firmer character since its birth, and was a different child in character and appearance than to what he should have found it, had he seen it at birth or shortly afterwards; but the general appearances in other respects, as well as in the last stated, confirmed the opinions and conclusions of its being a most decidedly premature child, conformable to the specific report already drawn up—the other appearances alluded to, were smallness of head, with roundness, divarication or separation of suture, small features generally, small arms and legs, nails developing and small, small downy hair, and general appearance of trunk feeble—the sexual organs, especially the nymphæ, more projecting; and the child appearing like one who had struggled with weakness, and was now commencing to improve."

Mr. Robert Menzies, factor and cashier for the Earl of Elgin, Fife, himself the father of eleven children, and upwards of fifty years of age, saw the child about two months after its birth. "It was a very 'smally' child in size, and he did not remember to have seen one at that age so small. He noticed particularly the smallness of its face, its wrists and hands, being all that was exposed; he never saw such small wrists, *they seemed to him not thicker than his little finger*."

Mrs. Anne Irvine, or Boswell, Dowager of Balmuto, widow of the late Lord Balmuto, with her daughter, visited the Manse at Kinghorn nine weeks after the birth of the child, and there saw it, when, from its extreme delicacy, she would have had great hesitation in handling it. It was both very small and had a great heaving at the breast, with quick difficult respiration.

Mrs. Jane Melville or Cook, of Kirkcaldy, saw the child in question three months and three weeks after its birth, and certainly thought it from its appearance a premature child, it was so very small—and, altogether, its skin and body seemed unlike those of any other child. The skin was not so full as it ought to be—but darker in colour, and to appearance like that of an old person. There was no hair on its head. And its heels seemed to her very imperfectly formed.

She had never seen a child born after less than six months' gestation, but has seen twins born in the seventh month, and the only difference between this child and them, was, that they died in a few hours, and it was certainly livelier.

The size of the child after death appears to have been smaller than the ordinary size of other children at their births—and that after a life of suffering protracted for several months. Its weight was not ascertained, certainly, by any of those witnesses, but guessed by more than one not to have exceeded three pounds, with its clothes on.

The conclusion of all the witnesses already specified, with the one only exception of Mrs. Robertson, herself the wife of one of the petitioners; or, in plain English, accusers; or, in plainer Scotch, libellers of the child's father, was that it was certainly the fruit of wedlock, lawfully begotten, and prematurely born. Mrs. Jardine's mother gives an additional reason for coming to such a conclusion, founded upon a fact of which she alone could be cognizant. "She had no doubt, from all she saw and observed at the time, that the child was born prematurely, and begotten in wedlock. It was, particularly in regard to the extremities, just as nothing, compared in size to its own mother, when born. *Mrs. Jardine herself having been a seven months' child.* It was so small in comparison."

The corpse of the infant was examined after its death by Dr. Reid, and Mr. Storrer of Kirkcaldy, within a day or two of its death. The following were the post-mortem appearances:—*Thorax.* Lungs, flabby in their texture and considerably engorged. The heart, smaller in size than the examiners thought it ought to have been at that particular age.—*Abdomen.* All the viscera sound.—*Head.* Not examined.

The legal and medical history of the case being before our readers, we have only to add the facts and opinions of the medical witnesses.

### III. FACTS ADDUCED BY THE MEDICAL WITNESSES IN CORROBORATION OF THEIR OPINIONS.

1, By Dr. ALEX. REID.—The size and appearance of the child at birth, being those of prematurity.

2. Mr. STORRER.—The case of another child, from his own practice, born at the end of six months' gestation, and which lived eight hours. It was very weakly, swallowed nothing, moaned a good deal but did not cry, the eyes were open, the skin soft and wrinkled, nails imperfectly formed. It was neither weighed nor measured, but was very small. *The mother had previously had a child, but one still-born, to the same husband.*

3. Professor HAMILTON.—His *negative experience* of 49 years' practice as an accoucheur—and his *positive unbelief* of every other practitioner whose experience differs from his own.

4. Dr. CAMPBELL.—No such production as a viable child, born at less than seven calendar months' gestation, being consistent with his *experience*, during a period of 22 years' practice ; or with his *knowledge*, embracing as that does, either directly or indirectly, upwards of 8000 deliveries.

Has, nevertheless, seen a child of four months and a half live a few minutes ; a child of six months' gestation merely gasp when born, and die immediately ; another live twenty minutes, and another three days.

5. Mr. ZIEGLER.—His *negative* experience of five years' practice as an accoucheur. His *positive* ignorance of any sufficiently authenticated case of a viable child born at less than seven calendar months' gestation.

6. Dr. Fairbairn.—The experience of 19 years; and acquaintance with 2,000 midwifery cases, including "a good many" immature children.

7. Professor Thatcher.—Personal experience as an accoucheur for thirty-two years, and in about 10,000 cases of midwifery. Personal examinations of the infant in question, of the attendant accoucheur, of the recipient witness to its birth, and of its nurse. Cases, in his own practice, of children born after a shorter period of gestation than seven months, and living—especially "three remarkable cases of children (in one year, 1835) born decidedly before the completion of seven months of utero-gestation or pregnancy," and alive May 3rd, 1838. These children were born, on or about the sixth month—bearing the following characters of immaturity, viz.:—diminutive development; small head and extremities; nails slightly developed; little hair, and that little downy; "a peculiarly corrugated brow when crying;" the face, that, denominated by the French, "*le petit vieillard*;" generally whining and discontented; inability or but partial ability to suck; requiring to be fed very slowly and gradually; *a peculiar appearance of the eye, in regard to the membrana pupillaris, which in these cases was just disappearing*; length and weight correspondingly short and small, the former being from twelve to fourteen inches, the latter from three pounds two ounces to three pounds five ounces; sexual organs peculiar in appearance, the nymphæ in the females being fuller and more protuberant, and the testes of a male beginning to descend; affected by icterus, or "yellow gum," as jaundice in young children is popularly denominated. The mothers of all three children were married—living with their husbands—and of the most respectable and undeniable character. One case was that of a second child—another, that of a second or third child—and the third was born of a mother who had had several children.

8. Mr. Millar.—A case occurring in his own practice, of a female child where he had every reason to believe that the gestation did not exceed six calendar months; and was alive at the time of his deposition, a month after the birth. The parents had been known to him as a married couple for at least five or six years; and had had other children.

9. Professor Buchanan.—One case of birth, six calendar months and three days from the last appearance of the menstrual discharge; the child survived, and when Dr. B. left America thirty years after, was serving there as a lieutenant of Marines. And one, which occurred in his own practice, of a child at 191 days, which died the day after its birth. *The lady had been married for years, and had had several abortions.*

10. Dr. Combe.—One case, positively spoken to, in which he considered the birth to have taken place from eight to fifteen days short of the completion of seven months. The child lived, to the Dr's. knowledge a year afterwards, and then he lost sight of it, and could not tell whether still alive or not. The mother was married, cohabited with her husband, and had had three or four children previously.

11. Dr. Macwhirter.—A case in his own family, occurring at the 190th day of gestation, calculating from the day after the catamenia had been last seen. The child was his own son, born at Calcutta, and alive at the time of the trial, 18 years and a half old. The birth had been brought on apparently by mental agitation.

12. Mr. Aitkin, Surgeon.—The case of a Mrs. Younger, at Coaltown of

Callange, visited by him in August, 1835. A mother already and a married woman, living and cohabiting with her husband, and who calculated that, to the best of her knowledge, she was between four and five months gone with child at the time of his visit. On the 12th of September following, she was delivered of a living female child. On the 17th died, and was survived by her child five weeks and one day. It measured thirteen inches, and weighed about three pounds. Its skin was yellow and much shrivelled. Its voice, feeble. And the only cause to which he could trace its death, was its removal to a very cold house.

13. Professor Alison.—Two cases by Belloc, of children born as early as the sixth months, and reared. One by Dr. Rodman, of Paisley, Edinb. Med. Journal, vol. xi., of a child not five months, which must have been very premature, "for it seemed to be kept alive only by its being wrapped in cotton, and kept close to its mother or to another woman; it could not suck at all for three weeks after birth, and when three weeks old, did not weigh two pounds, whereas the average weight at birth is nearly seven pounds, and very few live that are only three pounds weight at birth, yet this child was alive and thriving at the age of four months." And three other cases recorded by Dominico Meli, on good authority, of children believed to have been born at the fifth month, or very soon after, and which lived.

14. Dr. Reid.—Belloc's cases, already cited:—two cases related by Schencke, two by Velpeau. The case of Mr. Crib, that of Mr. Baker also. And especially Hencke's case, given on the authority of Dr. Outrepont, professor of midwifery at Wurtzburg.

15. Professor Christison.—Three cases, one related by *M. Belloc* in his Cours de Médécine Légale; another, Dr. Rodman's, referred to twice already; and the third, by Dr. Outrepont, published by Hencke in his valuable periodical, the Teitschrift für die Straats-arzneikunde. This last, especially, as "the only quite unequivocal instance hitherto published, of the rearing of a six months' child." "*The evidence is as complete as it is possible to be in any case of this kind.* It is complete, both as derived from the date of the mother's impregnation, and as drawn from the structure and history of the child. The mother, a young woman, whose catamenia had always been perfectly regular, was repeatedly connected with her husband, for some time after the cessation of their last flow. About a fortnight after this cessation of their flow, she underwent a general change in appearance, and began to have frequent attacks of vomiting and fainting, symptoms which she never had in her life before. These symptoms continuing, the catamenia did not return; and, about twenty weeks after their last appearance, she felt the first movements of the child. Five weeks after this, and twenty-seven weeks after the last appearance of the catamenia, she was seized with labour-pains and uterine hæmorrhage, upon which Dr. Outrepont, having discovered that the hæmorrhage proceeded from the placenta being attached over the os uteri, encouraged the labour, and brought it forthwith to a prosperous conclusion. Here, the evidence of the child being not more than 27 weeks, and possibly only 25 weeks old, is as strong as in the nature of things it is reasonable to expect. Every source which can supply any criterion of the accuracy of the reckoning is called into requisition. The state of the child at birth is even more unequivocal. It was a boy, and breathed immediately on being born. It measured 13½ inches, and weighed one pound and a half. Its skin was covered with smooth lank down, and was much wrinkled. The whole extremities were extremely small in proportion to the trunk, and were kept constantly bent over the body, as during the existence of the fœtus in the womb. The nails of the fingers and toes were like mere white folds of skin—the testicles were still within the belly, and the pupillary membrane was entire. The child whined, but could not cry—slept almost constantly—awoke only once a day—seldom opened its eyelids, and was obviously insensible both to light and sound. For some time it was fed with the spoon, on diluted milk and sugar. In four weeks the down began to drop off from the skin. In 15 weeks, it had made very little progress in any respect. The wrinkles had disappeared, however, from the skin, and the length was increased an inch and three quarters. But from this time, which corresponded with the 40th or 42nd week after impregnation—that is, with the full period of utero-gestation—it made rapid advances—sleeping less—eating more—crying strongly—and becoming evidently sensible to sound, and pleased with the light. When 14 months old, it was of the weight and stature of a child born at the full time. In the eighteenth month, the testicles descended into the scrotum, without causing him any annoyance. In like manner the teeth began to appear easily in his third year. He did not begin to walk till half a year later; and at that time he differed from other children of the same age, not only in littleness, but likewise in the singular oldness of his expression of countenance. When Dr. Outrepont saw him in 1816, he was eleven years of age, was as big as a boy of seven or eight, and had just begun to read and write."

In the concluding words of the learned Professor, to whom we are indebted for the case itself—it is "quite decisive of the possibility of rearing a child born at the end of the sixth solar month, or in 26 weeks."

### IV.—Opinions of the Medical Witnesses.

The *medical opinions* are, as in all such cases they have hitherto been, very discrepant. We shall not now notice the discrepancy, further than to observe, that in the present case it is partly accounted for by the very different amount of information respecting the particular case possessed by the witnesses for the prosecution, and those for the defence.

The opinions these gentlemen gave were founded partly upon their own experience, and partly upon the authority of others. We record them singulariter et simpliciter, as they were given in the order in which the witnesses were called before the Court.

1. Alexander Reid, M.D. surgeon, residing in Kirkcaldy; a practitioner of 22 or 23 years' standing.—*Decided*, that the child in question was prematurely born; and that the period of gestation at which a viable child may be produced is six calendar months.

2. James Storrer, surgeon in Kirkcaldy; a practising accoucheur for 13 years—deliveries accomplished by him averaging, during the latter years, three a week.—Seven months is the shortest period of gestation at which he has *known* a child to have been born alive, and capable of being reared.

3. Dr. James Hamilton, professor of midwifery in the University of Edinburgh since the year 1800; an accoucheur of 49 years' standing.—Has never known any infant born before the completion of six calendar months live, or rather exist above a few hours. In lecturing upon the subject, he makes a distinction between abortive infants and immature infants, and the result of his experience has uniformly been, that all infants born before the completion of seven calendar months after conception are abortive, that is, incapable of being reared. The shortest period of gestation after which a viable child may be produced is, at least, seven months. There is no difficulty, even the very smallest, to an experienced practitioner, in distinguishing between a child born before and after the expiration of seven calendar months. Places no credit in the cases recorded by foreign authors on subjects relating to practice which can only be investigated by persons of experience and veracity ! ! !

4. Dr. William Campbell, M.D. & F.R.C.S.E. residing in Edinburgh; a lecturer on midwifery in that city since 1819—a practising accoucheur since 1816—since when, has had either a direct or indirect responsibility in upwards of 8000 deliveries.—His belief is, that Mr. Jardine's child could not have been begotten after the marriage of its parents. Has known one child live ten or eleven days, who was born as nearly as could be calculated one week before the conclusion of seven calendar months. No child, which has been a shorter time than seven months in utero, can be reared. In the case of Madame Villars, reported by Desgranges, prefers the supposition of a double uterus to the admission of viability at so early a period as five months and sixteen days !

> Where others toil with philosophic force,
> Your nimble fellows take a shorter course ;—
> Fling at your head, conviction—in a lump,
> And gain remote conclusions at a jump !

5. Alexander Zeigler, Esq. surgeon in Edinburgh for the last 20 years, and a practising accoucheur there for the last five years.—The child in question could not have been begotten subsequent to the marriage of its parents :—having attained to, at least, seven calendar months of gestation. Has never, in his own practice, known a child reared, or even born alive, until after the completion of seven calendar months, which is the shortest period at which a child may be born, capable of being reared. The number of reported cases to the contrary do not appear to him sufficiently authenticated. Although some writers, both in this country and on the Continent, have alleged that infants have been reared when born at 4½, 5, 6, and 6½ months of gestation.

Although Velpeau has produced much analogical reasoning in favour of precocious births, it can only be settled by an appeal to experience.

6. Dr. John Thatcher, M.D. lecturer on midwifery in Edinburgh for the last 20 years. An accoucheur since Nov. 1806, in most extensive practice, attending during the earlier part of it from perhaps 300 to 500, and during the last 10 or 15 years from 200 to 300 deliveries annually.

Considered the child to be decidedly premature—and not older than alleged by its parents. And that it is quite possible for a child to be born and live, previous to the conclusion of the seventh calendar month. His own experience enabling him to state this.

"Interrogated, whether there is any anatomical or physiological change, which invariably takes place in the fœtus at a particular period of gestation within which it is impossible, but beyond which it is possible, for a viable child to be born? Depones, I consider this a very abstruse question, as so much must depend upon the phenomena in the development of the fœtus in utero, which may be contingent on various phenomena connected with the age and health of either parent, and particularly in connection with the immediate phenomena connected with its uterine life or existence; but at the same time, if he were to venture to give a probable opinion in connection with all these circumstances, he would humbly venture to state, that it is very unlikely for a fœtus or child to live previous to the fifth month or thereby of utero-gestation; but as children vary in size and development at the full period, it is equally licit, just and reasonable, to presume that there may be variations of development in the early as in the latter months of utero-gestations, for which all allowances are and must be made, rendering it thus impossible as presumptuous to fix a decisive period for the viability or non-viability, and rearing of an infant expelled from the womb." 97.

7. Alex. Millar, licentiate and fellow of the Royal College of Surgeons in Edinburgh. An accoucheur of eight years' standing, with the experience of from 800 to 1,000 deliveries. Is of opinion that a viable child is capable of being produced "about the sixth month of utero-gestation."

8. Dr. Walter Buchanan, residing at Mount Pleasant, Greenock. A graduate of Glasgow, in general practice in America from 1799 to 1806; then, professor of midwifery in Columbia College, New York, afterwards, in 1812, a naval hospital surgeon under the American government. Does not consider it impossible that a child capable of being reared should be born after a gestation of 174 days. When asked if he considered it probable, he answered:—"There is a great difference between possibility and probability, and a great deal would depend upon the physical development of the child."

9. Dr. James Scarth Combe, Physician in Leith, and F.R.C.S.E. seventeen years in the practice of midwifery, with an annual average latterly of 100 deliveries; does not think it impossible for a child born after a shorter period of gestation than seven full calendar months, to be reared. And is unaware of any reason, anatomical or physiological, which should render it impossible for a viable child to be born after a gestation of 174 days. Believes no child to be viable before the completion of the fifth month.

10. Dr. John Macwhirter, physician and accoucheur, residing in Edinburgh. Does not think it impossible that a viable child should be born before seven full calendar months. Doubts of the viability of any child, if born upon the 153rd day of utero-gestation. But thinks a viable child might be produced after having been in the womb 26 weeks.

11. Dr. William Pultney Alison, physician in Edinburgh, formerly professor of medical jurisprudence in that University, at the time of his examination president of the Royal College of Physicians, and one of the clinical physicians to the Royal Infirmary. Without any practical experience as an accoucheur, never having practised in midwifery. Adheres to the opinion expressed in his notes of lectures, that the possibility of a child born at or even before the end of the sixth month, living, is not to be denied—knowing nothing in the structure of a fœtus about the age of six months, which should make its maintenance of a separate existence incredible. While he draws only the negative inference, "that he cannot deny the possibility of a child living at this early period, he would say farther, that he thinks it on the whole probable, that there are instances, though very rare, of children born between the fifth and seventh months living at least some months."

"Interrogated:—Whether the deponent is aware that there are high authorities holding that a child is not viable till about the completion of the seventh month of gestation? Depones,—That he is perfectly aware of that, but does not consider it a question to be decided by authority, unless these authors had given a reason for thinking it impossible, and therefore for setting aside the opinion which he before gave, that it is a question to be decided only by fact and testimony. Interrogated:—Being a question to be decided by fact and testimony, does the deponent not consider that, if he had himself had an extensive practical experience as an accoucheur, he would have been better able to form a satisfactory opinion on the subject than he can be without that experience? Depones, —That he does not think that extensive practice as an accoucheur would have enabled him to weigh better the testimony of different authors, upon which alone, as he thinks, in the present state of our knowledge, the question can be decided. He might have seen 5000 children born, and not one live below the 7th month, but this would not have entitled him to conclude that none can live below that month. He might have seen 5000 children born, and not one live that was below the weight of three pounds, but all that experience would have weighed for nothing against the fact, which no such experience is necessary to enable him to state, that children have lived whose weight when born was below two pounds."

12. Dr. John Reid, physician and lecturer on medicine in Edinburgh, is of opinion that it is possible for a viable child to be born after a gestation of 174 days. Believes that no one can with safety fix any period of gestation within which it is impossible, but beyond which it may be possible, for such a child to be produced.

13. Dr. Robert Christison, professor of materia medica in the University of Edinburgh since 1832, and eleven years before that date professor of medical jurisprudence. No body doubts, now-a-days, the possibility of utero-gestation being prosperously concluded in less than nine solar months, or 280 days. The birth of a child at the 8th month, who subsequently attains the age of manhood, is far from being an uncommon occurrence. The possibility of a child capable of living, being born at the end of the 7th solar month is universally admitted; at the same time the rearing of such an one is a matter of much difficulty. The possibility of rearing a child born at the end of the sixth solar month is decisively established by Outrepont's case, and when to it is added "the other published cases which supply presumptive evidence to the same effect, we must, I think, admit that such an incident, in all probability, happens often enough to deserve being kept in view, in legislative enactments." Is of opinion that it is not impossible for a viable child to be born after a gestation of 174 days.

It is clear, that, so far as authorities go for any thing, their weight very greatly preponderates in favour of the prematurity of Mr. Jardine's child. The majority of the facts adduced by the witnesses have been a long time before the medical public, but scattered through many publications, most of which are unknown, and some inaccessible to the majority of readers. We have already noticed them in a former part of this article; along with several additional cases of abbreviated utero-gestation brought together in the "Record of the Proceedings" we have been analysing—and for further details we must refer to the report itself should that ever be republished, which it well deserves, not only on account of the importance of the principle involved, but also on account of the exceedingly valuable information embodied in the evidence of most of the professional witnesses —whose high professional characters are fully sustained by their testimony on this trial, with only one or two exceptions, which from motives of delicacy we abstain from particularizing.

Our own deliberate conviction is, that the majority were fully borne out in the conclusions to which they came. That the General Assembly was fully justified in restoring to Mr. and Mrs. Jardine their character untarnished and pure. And that the philosophical opinions of Drs. Alison and Christison will sooner or later become the rule with the profession, viz:—that a viable child may be born between the fifth and seventh months of utero-gestation.

---

